# Oral Metastatic Undifferentiated Pleomorphic Sarcoma: A Case Report 

**DOI:** 10.22038/IJORL.2022.61622.3118

**Published:** 2022-09

**Authors:** Saede Atarbashi-Moghadam, Ali Lotfi, Shaghayegh Dowdani

**Affiliations:** 1 *Department of Oral and Maxillofacial Pathology, School of Dentistry, Shahid Beheshti University of Medical Sciences, Tehran, Iran. *; 2 *Graduated Student, School of Dentistry, Shahid Beheshti University of Medical Sciences, Tehran, Iran.*

**Keywords:** Cancer, Jaw, Metastasis, Oral cavity, Soft tissue sarcoma

## Abstract

**Introduction:**

Oral metastases are rare; nevertheless, they must be considered in the differential diagnosis of lesions in patients with a previous history of malignancy or older adults. The clinical signs of oral metastasis typically comprise pain, dysphagia, ulceration, bleeding, and paresthesia. Soft tissue sarcomas tend to affect the extremities and retroperitoneum. The most common metastases in the oral cavity are carcinomas, and sarcomas rarely metastasize to this area. Undifferentiated pleomorphic sarcoma (UPS) is a mesenchymal malignancy that infrequently affects the head and neck site. It shows a male predilection and occurs in all age groups. The lung is the most common area of distant metastasis in undifferentiated pleomorphic sarcoma.

**Case Report:**

This report presents a 61-year-old female patient with a painful bluish-purple mass of the posterior right mandibular alveolar mucosa. She had a history of thigh UPS about four years ago. An incisional biopsy was performed, and the specimen was stained with hematoxylin and eosin. Immunohistochemical antibodies for CK, S100, desmin, myogenin, MDM2, SOX10, and caldesmon were negative and focally positive for CD68. Ki-67 index was about 70%.

**Conclusions:**

This report aimed to increase awareness of a rare lesion by describing the clinical, histopathologic, and immunohistochemical findings of metastatic undifferentiated pleomorphic sarcoma of the oral cavity.

## Introduction

Sarcomas are rare malignant tumors with aggressive behavior originating from mesenchymal cells (1). 

Soft tissue sarcomas can occur anywhere in the body; however, the most common site is the extremities and retroperitoneum. The most common microscopic subtypes are liposarcoma, pleomorphic sarcoma, leiomyosarcoma, rhabdomyosarcoma, and synovial sarcoma. Metastasis of sarcoma is mainly hematologic, and approximately 90% of distant metastases occur in the lung. In sarcomas’ of extremity, metastasis occurs in about 25% of cases, especially if the primary tumor is deep and close to the fascia (2). 

Despite advances in immunohistochemical (IHC) staining and molecular analysis, the classification of sarcomas is still challenging, with approximately 20% of all sarcomas being unclassified (1). More than 50 microscopic subtypes have been identified for soft tissue sarcomas. Though each subtype shows a morphologic and anatomic distinction, their distant metastasis location is mainly kept to the lung. A few cases may also metastasize to lymph nodes (2). Oral metastatic neoplasms are rare, though they must be considered in the differential diagnosis of lesions in patients with a previous history of malignancy. The gingiva and tongue are common sites of metastasis to the oral soft tissues (3). They are more usually found in older adults (4).

Metastatic sarcomas are rare in the oral cavity; carcinomas are the most common metastases. Although oral metastases have a poor prognosis, the timely diagnosis of these malignancies is essential to the patient (5). Undifferentiated pleomorphic sarcoma (UPS), previously identified as malignant fibrous histiocytoma (MFH), is a mesenchymal neoplasm that seldom affects the head and neck area (6). Here we described the clinical, microscopic, and IHC findings of metastatic UPS of mandibular alveolar mucosa affecting a 61-year-old woman.

## Case Report

A 61-year-old woman presented a painful mass of the posterior right lower jaw. The extra-oral examination was normal, whereas she mentioned the pain in the chin area and lip paresthesia. There was no cervical lymphadenopathy. 

**Fig 1:**
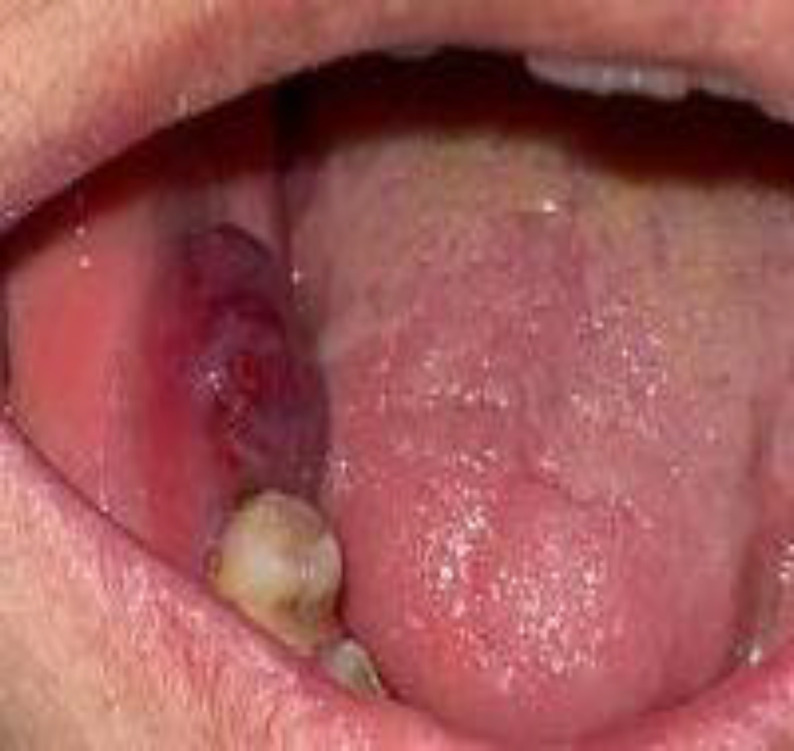
Photograph shows a bluish-purple mass covered by mucosa extended from the distal of the right second premolar

The hematologic examination was normal except for a low platelet count (129000 10^*3/µl^). ESR was within the normal range (11 mm/hrs), and CRP was 3+. Intraoral examination showed a painful bluish-purple mass covered by mucosa with soft consistency extended from distal of right second premolar to retromolar pad region measuring about 2cm×1cm with two months duration (Fig 1).

**Fig 2:**
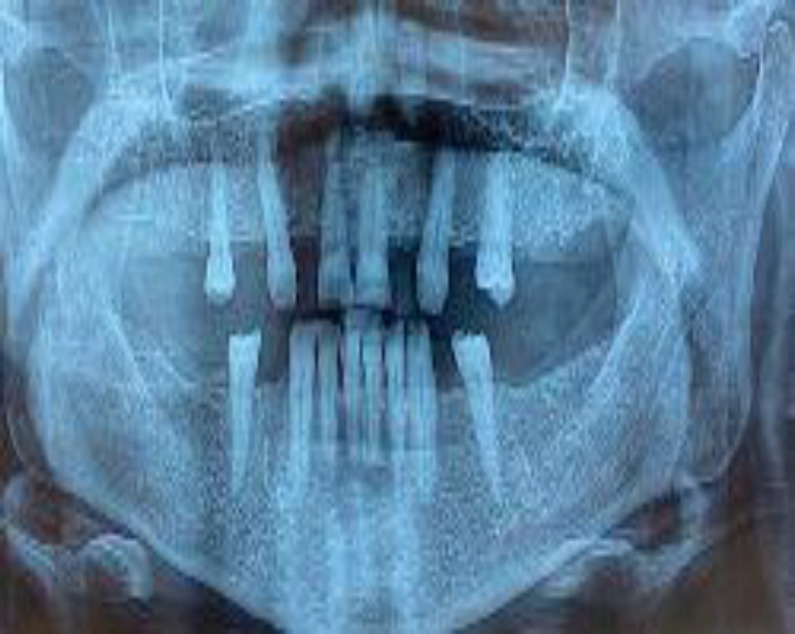
The mandibular bone was intact in panoramic radiography

The jawbone was intact in panoramic radiography (Fig 2).

**Fig 3:**
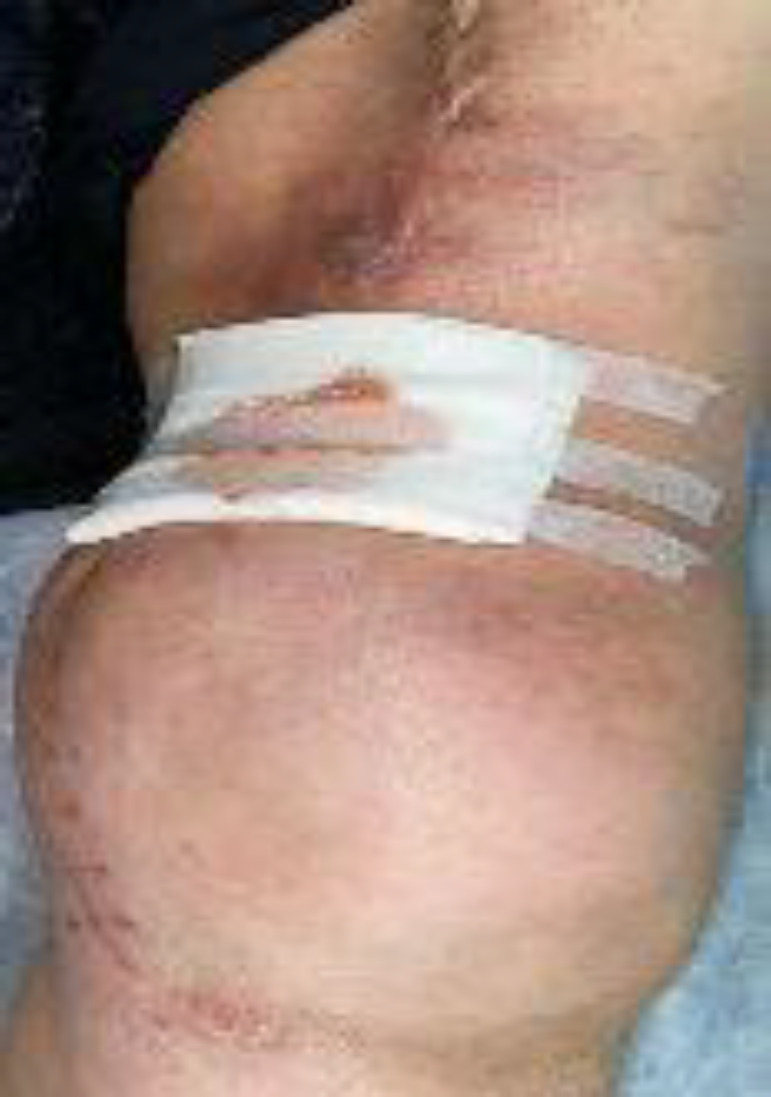
Photograph of the thigh after the tumor recurrence

She had a history of recurrent thigh UPS about four years ago, undergoing extensive surgery and radiotherapy (Fig 3). 

**Fig 4:**
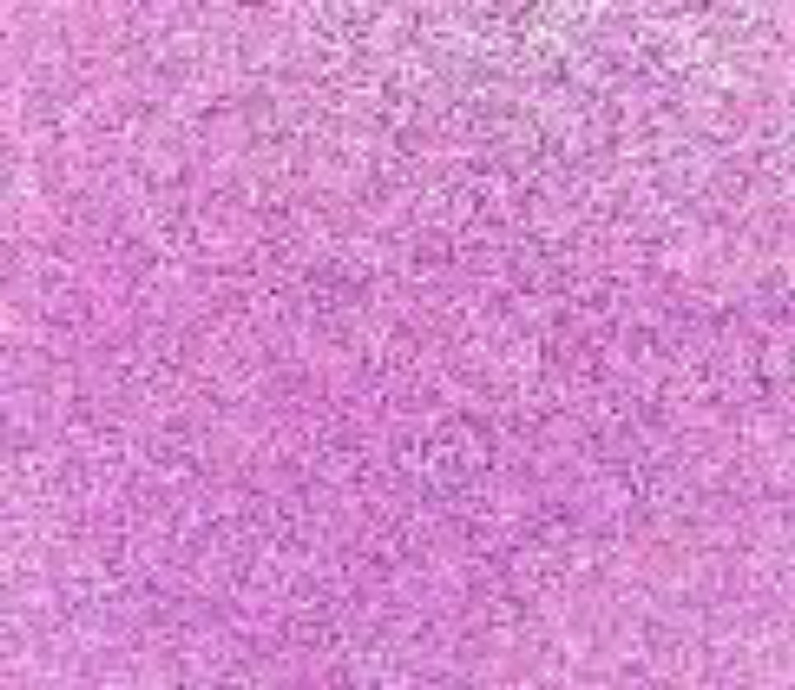
Microscopic sections show sheets of atypical spindle and some epithelioid cells with pleomorphic nuclei (×100)

Due to her medical history, the provisional diagnosis of the metastatic tumor was made and an incisional biopsy was performed under local anesthesia. Evidence of bone involvement was seen during surgery. On gross examination, the lesion was fragile and brown like granulation tissue with soft to elastic consistency. Microscopic examination showed a malignant mesenchymal neoplasm composed of sheets of atypical spindle and some epithelioid cells with pleomorphic and hyperchromatic nuclei. In addition, giant tumoral cells, many mitotic figures, and foci of necrosis were also seen (Fig 4).

**Fig 5:**
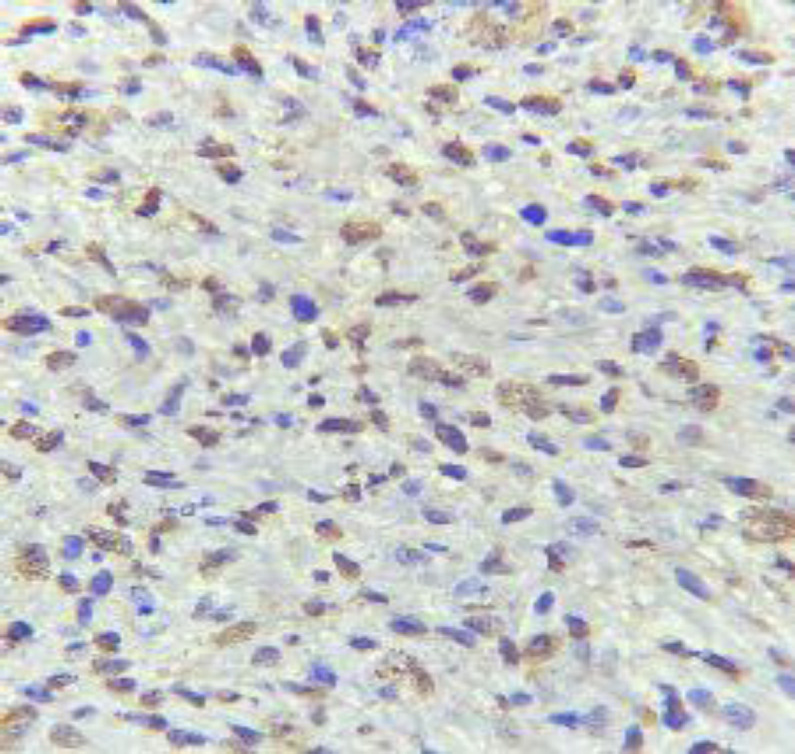
Ki-67 index about 70% (immunohistoche- mistry, ×400).

The primary tumor slides were requested, and the microscopic appearance was consistent with the current oral lesion. Immunohistochemistry (IHC) for CK, S100, desmin, myogenin, MDM2, SOX10, and caldesmon was negative and focally positive for CD68. Ki-67 index was about 70% (Fig 5).

Based findings mentioned above, a diagnosis of metastatic UPS was made, and a whole-body scan was recommended to identify other metastatic lesions. Unfortunately, the patient died of Covid-19 infection before performing a whole-body scan and completing treatment.

## Discussion

Soft tissue sarcoma metastasis to the oral cavity is exceedingly rare, with only a few cases reported in the literature (2,3,5-7). One of the most common sarcomas that metastasize to the oral cavity is leiomyosarcoma (2,5,8-11). To the best of our knowledge, there is no report of oral metastasis of UPS. However, several cases of MFH and undifferentiated spindle cell sarcoma metastasizing to the oral cavity have been reported (12,13,14). Metastasis to the oral cavity typically describes widespread lesions (3). The clinical signs of oral metastasis usually comprise pain, dysphagia, ulceration, and bleeding (2). 

Our patient also suffered from a painful mass with lip and chin numbness. A numbed chin is a significant diagnostic symptom of metastatic lesions (3). In dentate cases, 80% of metastases are seen in the gingiva, while, in edentulous patients, metastases are divided between the tongue and alveolar mucosa. In the current case, alveolar mucosa involvement was also seen. The presence of teeth is essential in the tendency of metastasis to the gingiva. The rich vascular network of inflamed gingival tissues may be a source of nutrition for the further growth of metastatic cells (3). The lung is the most common area of distant metastasis in UPS, and in the head and neck region, metastasis is rare (6). UPS shows a male predilection and occurs in all age groups (15). 

Kaplan et al. (16) mentioned that in older adults, metastatic lesions need to be comprised in the differential diagnosis of oral masses at any oral site, whether or not a specific primary neoplasm has been reported. Pleomorphic sarcomas are a diverse group of mesenchymal neoplasms with broadly different clinical behavior but overlapping microscopic features. Some of them, like Myxofibrosarcoma, USP, and Pleomorphic leiomyosarcoma, show a tendency for somatic soft tissues, particularly the thigh. Others often occur in the retroperitoneum, such as dedifferentiated liposarcoma and pleomorphic leiomyosarcoma, (17). They show a marked difference in the pattern of recurrence and metastasis. For example, pleomorphic myogenic sarcomas demonstrate very high metastatic potential, and dedifferentiated liposarcoma has significant potential for local recurrence. It is crucial to exclude metastatic sarcomatoid carcinoma and melanoma before diagnosing a pleomorphic sarcoma (17). 

In the current case, local recurrence, lymph node involvement, and distant metastasis were observed. UPS is a diagnosis of exclusion. This neoplasm does not have distinct histological characteristics and may reveal a fascicular, storiform, or sheet-like proliferation and frequently shows an admixture of spindle and pleomorphic, rarely multinucleated cells, with palely eosinophilic or slightly basophilic cytoplasm (17). The present case also showed sheets of spindle, epithelioid and tumoral giant cells with a high mitotic rate (≥60%) and necrosis in primary and metastatic tumors. If a metastatic tumor is suspected, a microscopic examination of the primary lesion slide and its histopathologic resemblance to a metastatic lesion is helpful (5,18). A cytogenetic examination is not valuable in most cases of pleomorphic sarcoma. Diagnostic markers include SMA, desmin, and caldesmon for pleomorphic leiomyosarcoma, desmin, myogenin, and MyoD1 for pleomorphic rhabdomyosarcoma, and MDM2 and CDK4 for pleomorphic liposarcoma. In addition, Keratins, S100 & SOX10 are used to rule out sarcomatoid carcinoma and melanoma (17). Hornick (17) reported that expression of CD34 and focal SMA might be seen, as nonspecific. In the current case markers, such as CK, S100, desmin, myogenin, MDM2, SOX10, and caldesmon were negative, and only CD68 was focally positive. Complete surgical resection is the treatment of choice for primary UPS, and a 5-year metastasis-free survival rate is about 30%. Adjuvant radiation and chemotherapy are used as well (6). In the present patient, despite extensive surgery, lymph node dissection, radiotherapy, recurrence, and metastasis to the oral cavity were developed within four years, indicating a poor prognosis of this tumor.

## Conclusion

Metastatic sarcoma to the oral cavity is a rare phenomenon, with few cases reported in the literature. However, the possibility of a metastatic lesion should be considered in patients complaining of paresthesia (numb chin syndrome). Familiarity with these lesions' clinical and microscopic characteristics is critical for clinicians and pathologists to have a proper and timely treatment plan for the patients. 

## References

[1] Atarbashi-Moghadam S, Emami Razavi AN, Salehi Zalani S (2019). Prevalence of Head and  Neck  Sarcoma in a Major Cancer Center in Iran- A 10-Year Study. Iran J Otorhinolaryngol..

[2] Dehal A, Quach L, Garrett E, Jreije K, Hussain F (2015). Soft tissue sarcoma with tongue metastasis: A case report and literature review. J Oral Maxillofac Surg..

[3] Shah D, Shetty S, MacBean AD, Olley SF (2010). Numb chin syndrome: a metastatic deposit in the mandible. Dent Update..

[4] Gholami S, Bakhshi M, Atarbashi-Moghadam S, Mir Mohammad Sadeghi H, Rahimzamani (2020). Mandibular Metastasis of Silent  Papillary  Thyroid Carcinoma: A Rare Case Report with Review of the Literature. Case Rep Dent.

[5] Allen CM, Neville B, Damm DD, Marsh W (1993). Leiomyosarcoma metastatic to the oral region Report of three cases. .Oral Surg Oral Med Oral Pathol..

[6] Ramsey JK, Chen JL, Schoenfield L, Cho RI (2018). Undifferentiated Pleomorphic Sarcoma Metastatic to the Orbit. Ophthalmic Plast Reconstr Surg..

[7] Peters SM, Perrino MA, Yoon AJ, Philipone EM (2017). Alveolar soft part sarcoma metastatic to the mandible: A report and review of literature. J Stomatol Oral Maxillofac Surg..

[8] Hope I, Morton K, Newlands C, Butler-Manuel S, Madhuri TK (2017). Lockjaw from a metastatic uterine leiomyosarcoma- case report and review of the literature. BMC Womens Health..

[9] Azevedo RS, Pires FR, Gouvêa AF, Lopes MA, Jorge J (2012). Leiomyosarcomas of the oral cavity: report of a radiation-associated and a metastatic case. Oral Maxillofac Surg..

[10] Cassoni A, Terenzi V, Bartoli D, Rajabtork Zadeh O, Battisti A, Pagnoni M (2014). Metastatic  uterine leiomyosarcoma in the upper buccal gingiva misdiagnosed as an epulis. Case Rep Oncol Med..

[11] Fernández-Barriales M, García-Montesinos B, García Reija F, Mayorga Fernández M, Saiz Bustillo R (2013). Metastatic leiomyosarcoma of the oral region from a uterine primary: a case report and review of the literature. J Oral Maxillofac Surg..

[12] Van Hale HM, Handlers JP, Abrams AM, Strahs G (1981). Malignant fibrous histiocytoma, myxoid variant metastatic to the oral cavity Report of a case and review of the literature. Oral Surg Oral Med Oral Pathol..

[13] Geist J, Azzopardi M, Domanowski A, Plezia R, Venkat H (1990). Thoracic malignant fibrous histiocytoma metastatic to the tongue and skin of the face. Oral Surg Oral Med Oral Pathol..

[14] Maschino F, Guillet J, Curien R, Dolivet G, Bravetti P (2013). Oral metastasis: a  report of 23 cases. Int J Oral Maxillofac Surg..

[15] Cambruzzi E, Cruz RP, Gava VG, Pêgas KL (2020). Undifferentiated high-grade pleomorphic sarcoma of the larynx treated with partial laringectomy. Braz J Otorhinolaryngol.

[16] Kaplan I, Raiser V, Shuster A, Shlomi B, Rosenfeld E, Greenberg A (2019). Metastatic tumors in oral mucosa and jawbones: Unusual primary origins and unusual oral locations. Histochem..

[17] Hornick JL (2018). Subclassification of  pleomorphic sarcomas: How and why should we care?. Ann Diagn Pathol.

[18] Atarbashi-Moghadam S, Lotfi A, Sijanivandi S (2022). Asymptomatic radiolucent lesion of the mandible. J Stomatol Oral Maxillofac Surg..

